# Evaluation of Clinical Graded Treatment of Acute Nonsuppurative Otitis Media in Children with Acute Upper Respiratory Tract Infection

**DOI:** 10.1155/2021/5517209

**Published:** 2021-04-02

**Authors:** Wei Meng, Dong-Dong Huang, Guang-Fei Li, Zi-Hui Sun, Shuang-Ba He

**Affiliations:** Department of Otorhinolaryngology Head and Neck Surgery, Nanjing Tongren Hospital, School of Medicine, Southeast University Nanjing, China

## Abstract

**Objective:**

To treat children with acute nonsuppurative otitis media induced by acute upper respiratory tract infection of varying severity and evaluate its therapeutic effects.

**Materials and Methods:**

Patients from the emergency department with acute nonsuppurative otitis media were followed up between September 2015 and December 2018. A total of 420 patients were classified into grades I to III according to tympanic membrane intactness and systemic reactions and treated according to grading.

**Results:**

Grade I patients showed no significant difference in the recovery of acute symptoms whether antibiotics are used or not. Grade II patients, after 3 months of follow-up, showed no tympanic membrane perforation, and 9 cases of binaural B-type children did not improve but were cured by operation. In grade III patients, after treatment for 4 hours in the experimental group 3, the earache subsided, 1 case had tympanic membrane perforation, and the patients recovered after 2 weeks (64/92) and after 3 months (28/92) of drug treatment. After treatment for 4 h in the control group 3, the earache eased, and 3 patients developed tympanic membrane perforation and were treated for 3 months. 4 binaural B-type children did not improve but recovered after surgical treatment.

**Conclusion:**

Grade I patients could be closely followed up by clinical observation. For anti-inflammatory patients with grade II disease, treatment has therapeutic significance. For patients with grade III, some patients still have TMP, but the use of cephalosporin third-generation drugs plus an appropriate amount of hormone therapy is effective in reducing symptoms and tympanic local reactions.

## 1. Introduction

Acute otitis media (AOM) is one of the most common diseases in children in the otolaryngology department. AOM is an infection of the mucosa in the middle ear cavity caused by bacteria and/or viruses, directly entering the tympanum through the eustachian tube and is usually followed by a common cold [1]. AOM is more common in children between the ages of 6 months and 3 years. The three most common bacterial infections are *Streptococcus pneumoniae* (25-50%), *Haemophilus influenzae* (15-30%), and *Moraxella catarrhalis* (3-20%). AOM is classified into acute nonsuppurative otitis media and acute suppurative otitis media. Acute nonsuppurative otitis media refers to the tubal pharynx, mouth, and cartilage segments, inflammatory mucosal hyperemia, swelling, and congestion after acute upper respiratory tract infection and may be accompanied by bacteria or viruses via the eustachian tube, directly into the middle ear cavity, resulting in an inflammatory reaction in the middle ear mucosa. In the early acute inflammatory stage, it is accompanied by changes in the tympanic membrane and inflammatory serous or mucous exudation changes in the middle ear cavity in the late stage. The disease often coexists with upper respiratory tract infection and can include recurrent attacks, sudden onset, and one or more symptoms. The symptoms of acute nonsuppurative otitis media are mainly local symptoms, such as ear pain which is persistent and can be characterized by irritability, sometimes covering and pulling ears, most often affecting sleep. Only those having an upper respiratory tract infection in the early stage can present with fever. Signs seen in the early stage are mild congestion, depression of the tympanic membrane, and deformation of the light cone. Tympanic effusion shows a dull, pale yellow, or amber, tympanic membrane sometimes with an arc-shaped liquid level line. Acute suppurative otitis media refers to its pathological change, which is caused by a large amount of middle ear exudate formed by the negative pressure of the middle ear in the early stage, which becomes the culture medium of bacteria, leading to continuous invasion of the suppurative bacteria through the eustachian tube, resulting in high reproduction rate, responsible for the absorption of toxins causing systemic fever symptoms. Its pathological manifestations are congestion and swelling of middle ear mucosa, increased purulent secretion, hyperemia and convex tympanic membrane, and even perforation and purulent discharge. Intracranial and extracranial complications may occur if the infection involves suppuration of the mastoid cavity without timely drainage. In addition to the symptoms of local persistent ear pain and infant earache, the symptoms may also be accompanied by systemic symptoms such as high fever, crying, nausea, and vomiting, and the symptoms will be relieved after purulent ear discharges. Some children experienced early hearing loss. Symptoms include enlarged and convex tympanic membrane hyperemia areas, disappearance of tympanic membrane sign, rupture of tension part, perforation, pus overflow, and sometimes redness behind the ears.

The treatment of acute otitis media in children is mainly through the application of antibacterial drugs combined with other symptomatic treatments. Indications for the use of antibacterial drugs for acute otitis media in children are suspected nonsuppurative otitis media and suppurative otitis media caused by bacterial infection, especially in severe cases (ear purulent or with high fever ≥ 39°C) and young children, who should be treated with antibacterial drugs in time [2–8].

The three common pathogenic bacteria in children with acute otitis media include Streptococcus pneumoniae, Haemophilus influenzae, and Moraxella catarrhalis. Oral amoxicillin is recommended as the first-line medication to effectively fight against moderately sensitive strains of penicillin, and the course of treatment is 7-10 days [6]. Alternatively, macrolide oral azithromycin, which has a high tissue concentration in the middle ear mastoid infection site, has a significant effect on intracellular bacteria such as nonclassified Haemophilus influenzae, with a short course of treatment, long action time, and good compliance and is also suitable for those allergic to penicillin drugs [7, 8]. If the above drugs are ineffective, second- or third-generation cephalosporins can also be selected.

Because the shape of the tympanic membrane is very important for the diagnosis of diseases and judging the severity of lesions, this study classified children's acute otitis media according to pain score and tympanic membrane shape. Patients with perforation and purulent tympanic membrane or accompanied by systemic symptoms are definitively diagnosed with acute suppurative otitis media, and patients with acute nonsuppurative otitis media were classified and treated individually in order to establish a reasonable threshold for emergency medical treatment and procedures for children with acute nonsuppurative otitis media, while focusing on assessing the eustachian tube function and reducing complications.

The inclusion criteria, based on China's “Guidelines for the diagnosis and treatment of otitis media in children” 2015 diagnostic criteria, were as follows: (1) age 2-14 years, (2) 48 h within a sudden occurrence, (3) earache, (4) tympanic membrane intact with acute congestion, (5) middle ear effusion, and (6) a history of upper respiratory tract infection before onset.

The exclusion criteria were as follows: (1) tympanic tube and cochlear implants, (2) external ear canal redness or pus, (3) congenital head deformity, and (4) other serious infections, such as renal insufficiency, heart failure, malignant tumors, immune dysfunction, or other serious diseases.

## 2. Subjects and Methods

### 2.1. Subjects

The subjects of this study were children with acute nonsuppurative otitis media (2-14 years old, 208 males and 212 females) with an average age of 4.12 ± 2.31 years (Table 1).

This study included 420 cases: 293 cases of single ear, 127 cases of both ears, 106 cases of hernias, and 10 cases of recurrent episodes (repeated more than three times). These cases were classified into grades I to III based on the tympanic membrane intactness and systemic reactions.

### 2.2. Grading Criteria: Severity Grading of Acute nonsuppurative Otitis Media

Grade I: the tympanic membrane is mainly in the hammer bone, a small tympanic membrane, no effusion, normal shape, pain score of 4–6 points, and transient attack.

Grade II: tympanic membrane congestion is obvious, involving most of the tympanic membrane, no effusion, the shape is normal, a pain score of 6–8 points, and a transient attack.

**Figure 1:**
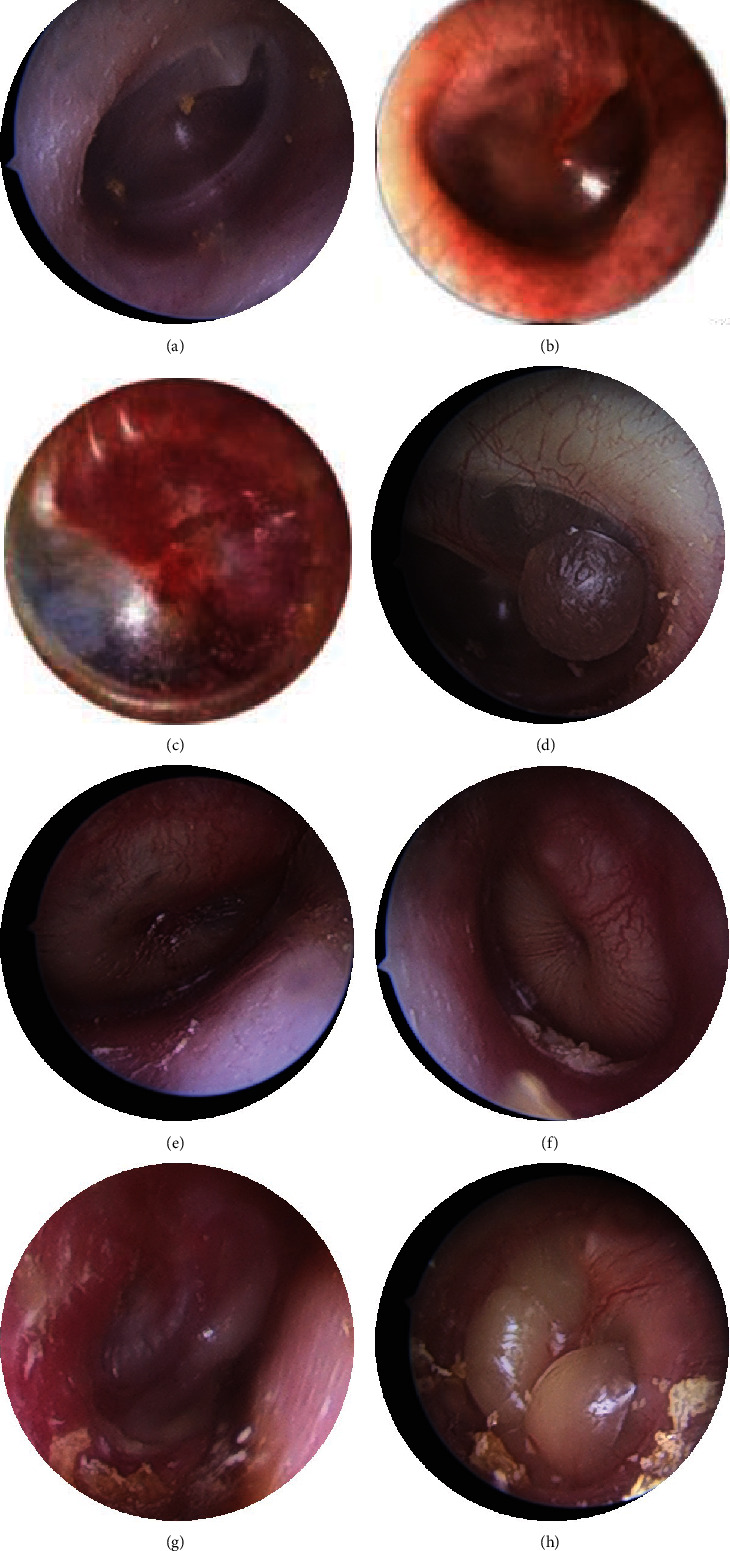
Grading criteria: severity grading of acute nonsuppurative otitis media: (a) normal, (b) grade I, (c) grade II, and (d)–(h) grade III.

Grade III: tympanic membrane congestion, effusion or empyema, obvious bulging of the tympanic membrane tension, pain score higher than 8 points, children with persistent ear pain, ear nausea, and symptoms may be accompanied with fever (Figure 1).

There were 147 cases of grade I, which were randomized into two groups: experimental group 1, which was administered oral antibiotics for 3 days (61 cases) and control group 1 without antibiotic administration (86 cases). There were 150 cases of grade II, which were randomized into two groups: experimental group 2 with oral antibiotics for 5–7 days (69 cases) and control group 2,with analgesic therapy which did not use antibiotics (81 cases). There were 123 cases of grade III, which were randomized into two groups: experimental group 3, which received a course of three-generation cephalosporins with appropriate steroid hormone by intravenous drip for 1 day (92 cases) and control group 3, who received oral antibiotics for 5–7 days (31 cases) and analgesic therapy.

### 2.3. Selection Criteria for Anti-Inflammatory Drugs

Based on common bacterial infections, third-generation cephalosporins are considered more sensitive, and the side effects are also reduced. For glucocorticoids, it is recommended to use sodium methylprednisolone succinate for injection (based on the weight of the patient, 1-2 mg/kg).

### 2.4. Efficacy Evaluation

Two weeks after the patient's acute symptoms subsided, the tympanogram was reviewed; if the tympanogram was type A, the treatment was terminated; if the tympanogram was type B or C, the patient was transferred to the late follow-up treatment (Table 2).

### 2.5. Late Follow-Up Treatment

**Figure 2:**
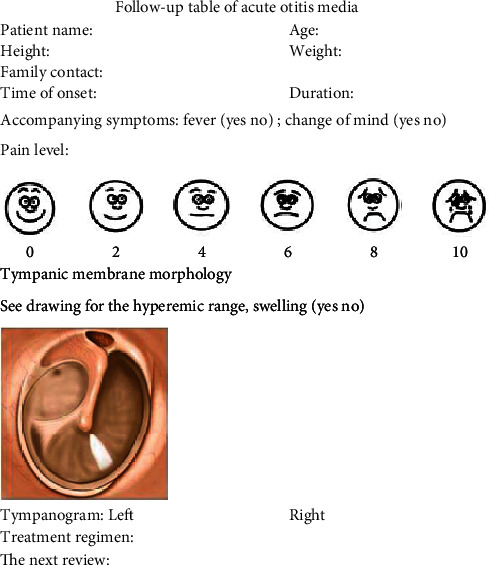
Clinical follow-up table of acute otitis media.

According to the situation, the corresponding treatment was followed up for treatment review. After a three-month conservative treatment (nasal hormone and oral discharge), the patient was observed for recurrence, adenoid hypertrophy, and swelling of the eustachian tube and pharyngeal mucosa, then treated by adenoidectomy and tympanic tube implantation, if warranted by the observation (Figure 2).

### 2.6. Statistical Methods

The data were statistically analyzed using SPSS software (version 19.0), and the measurement data were expressed as mean ± SD. The comparison between groups was made using the paired *t*-test and Chi-square test. Statistical significance was set at *P* < 0.05.

## 3. Results

### 3.1. Grade I

In 147 patients with grade I, the symptoms of earache in the experimental group 1 and control group 1 were transient, and there was no significant difference in the recovery of symptoms. 34 children had both ears of C-type, and nasal symptoms were treated with nasal hormones. In the last month, both groups were converted to type A; patients in class I were treated with antibiotics. There was no significant difference in clinical outcomes.

### 3.2. Grade II

Children with grade II (150cases), experimental group 2 (69 cases), and control group 2 (81 cases) were treated with no perforation of the tympanic membrane and children with sputum; there was no significant difference in pain relief and control.

Among the children with grade II (43/150) both ears type B, (31/150) binaural type C, and 9 cases of children with binaural type B after 3 months of standardized follow-up treatment were treated with surgery and the remaining patients recovered. In the experimental group, 23 patients with a b-tympanogram before treatment were treated, and 1 patients were treated. In the control group, 20 patients had a b-tympanogram before treatment and 8 patients after treatment. Apply the Chi-square test (*t* = 6.203, *P* < 0.05), there was a significant difference in negative pressure in the experimental group compared to the control group in the treatment of tympanic effusion (Tables 3–6).

### 3.3. Grade III

In grade III (123 cases), experimental group 3 (92/123) cases were treated with antibiotics and hormones once. Earache and ear swelling symptoms recovered quickly, and there was only 1 case of tympanic membrane perforation (TMP). After two weeks of tympanogram review, 64/92 patients recovered, and the remaining patients recovered after 1–3 months of conservative treatment. None of the patients underwent surgery.

In the control group 3, 31 of 123 patients were treated with antibiotics alone and 5 children with TMP. Ear pain and ear swelling symptoms partially recovered slowly. After two weeks of tympanogram review, 3/31 patients recovered, and the remaining patients recovered after 1–3 months of conservative treatment. 4 cases of B-type children with standard treatment for 3 months did not improve, and treatment was performed with adenoid radiofrequency ablation+tympanic membrane transplantation. After extubating, the patient healed (Tables 7 and 8).

There was a significant difference of the experimental group compared to the control group in the treatment of TMP, in the Chi-square test (*t* = 8.297, *P* < 0.05).

## 4. Discussion

AOM is a common and frequently occurring disease in children. The onset was sudden, and some lesions exhibited a self-healing tendency [9–11]. Clinical diagnosis is often performed in pediatric or otolaryngology head and neck surgery emergency departments, requiring more visits to the pediatric department [12–14]. Because of the lack of later clinical observation and follow-up, related research on the disease is gradually gaining attention from domestic otolaryngology head and neck surgery departments, with the main research focusing on the use of antibiotics. According to reports from the United States on the treatment of children with AOM and the self-healing tendency of AOM, observation treatment is under consideration [15–19].

In clinical observation, the tympanic membrane morphology is the gold standard for the diagnosis of AOM in children [20–25]. The researchers divided the clinically enrolled children with AOM into tympanic membrane morphology, accompanying symptoms, and children's crying degree and divided them into three grades. The corresponding treatments were then performed.

Conclusions from clinical studies were as follows:

1. The symptoms of earache in children with grade I were transient, even when antibiotic treatment was taken, and there was no significant difference in the recovery of acute symptoms. Some children had nasal congestion, and ear symptoms were treated with nasal hormones in the presence of nasal symptoms. Type A, whether antibiotic treatment was used or not, there was no significant difference in clinical effects; therefore, children with grade I can be observed, and no anti-inflammatory treatment is necessary
2. Children with grade II tumors should receive antibiotics to treat tympanic effusion. Negative pressure in patients treated with antibiotics was better than that in patients not treated with antibiotics. Children with grade II are recommended to receive oral antibiotics to strengthen the tympanic effusion for the treatment of negative pressure
3. Children with grade III disease should be treated immediately with hormones and anti-inflammatory agents. After earache, the relief of other ear swelling symptoms was obvious. These agents are known to have a certain effect in preventing perforation of the tympanic membrane. After a three-month follow-up and observation period, it was observed that anti-inflammatory and hormonal treatments have a significant effect on accelerating the regression of tympanic effusion in late stages

The investigators believe that the clinical treatment of acute nonsuppurative otitis media in children caused by an acute upper respiratory tract infection should be treated by evaluating the degree of severity, the absence of excessive intake of antibiotics for the treatment of low-grade disease, and insufficient treatment of more serious grades [26–30]. In case of incomplete remission, some patients may have prolonged symptoms, resulting in future hearing loss and cholesteatoma formation [31, 32]. Effective and not excessive treatment, clinical summarizing of the standardized treatment mode, convenient and quick referral modes, effective follow-up systems, and minimization of the impact of AOM on patients are the focus of this research. Through outpatient follow-up observation and appropriate treatment, the researchers improved the attention of the patient' s family members, reduced the occurrence of clinical symptoms caused by AOM, and treated the children in a timely manner, including those whose treatment was initially ineffective and required repeated treatment [33–37].

Given that clinical work requires special treatment, researchers carried out pediatrics and otolaryngology head and neck surgery and referred all patients with upper respiratory tract infection with acute ear pain to the otolaryngology head and neck specialist for grading, appropriate treatment program, and specialized disease treatment [38–40]. The process attempted to avoid shortcomings in the pediatric and otolaryngology departments by using clinical scales, speeding up the diagnosis, standardizing diagnosis and treatment processes, and facilitating scientific research data and follow-up of clinical treatment effects [41, 42].

The relationship between tympanic effusion in children with AOM and secretory otitis media is still not clear. These two diseases have complex connections in children. It is certain, however, that the two diseases mutually promote and create a vicious cycle therefore requiring close follow-up observations and treatment. Finally, the relationship between these two diseases is an important direction for future research.

## 5. Conclusions

This study showed no significant difference in clinical in 147 grade I patients regardless of antibiotic treatment. A total of 150 patients had grade II tumors that were treated with antibiotics for tympanic effusion. Negative pressure was significantly different in patients treated with antibiotics compared to those without antibiotic treatment. In 123 grade III patients who underwent hormone therapy, regardless of the ear pain after medication, the relief of ear swelling symptoms was obvious, and the perforation of the tympanic membrane was prevented. The acceleration of the regression of tympanic effusion was also observed in the late stage.

## Figures and Tables

**Table 1:** The basic situation of the researcher.

Gender	Quantity	Age	Height (cm)	Weight (kg)
Male	208	4.81 ± 2.29	99.8 ± 10.13	21.1 ± 3.14
Female	212	4.75 ± 2.27	99 ± 10.98	19.6 ± 3.08

**Table 2:** Efficacy rating.

Cure	All symptoms disappeared or the total score before and after treatment decreased by >80%
Better	Part of the symptoms disappeared or the total score decreased by 30%-80% before and after treatment
Invalid	No improvement in symptoms or a total score reduction of<30% before and after treatment
Aggravation	Increased symptoms or increased total score before and after treatment

**Table 3:** Pain score.

	Experimental group 2	Control group 2
Before treatment	7.2 ± 0.84	7.17 ± 0.76
After treatment	1 ± 0.71	2.4 ± 1.34
*t*	21.54	19.26
*P*	<0.05	<0.05

**Table 4:** Tympanic membrane morphology changers.

	Tympanic membrane congestion	Tympanic effusion
Experimental group before treatment	Serious	Presence
Experimental group after treatment	Lighten	Regression
Control group before treatment	Serious	Presence
Control group after treatment	Lighten	Regression

**Table 5:** The tympanic negative pressure of both ears before treatment.

	A	C	B
Experimental group	13	19	23
Control group	21	12	20

**Table 6:** The tympanic negative pressure of both ears after 3 months of treatment.

	A	C	B
Experimental group	44	10	1
Control group	41	4	8

**Table 7:** Pain score after 4 hours.

	Experimental group3	Control group3
Before treatment	9.34 ± 1.31	9.5 ± 1.30
After treatment	0.8 ± 0.84	2.4 ± 0.64
*t*	26.87	24.79
*P*	<0.05	<0.05

**Table 8:** Tympanic membrane morphology changers after 4 hours.

	Tympanic membrane congestion	Tympanic membrane dilating	Tympanic effusion
Experimental group before treatment	Serious	Serious	Presence
Experimental group after treatment	Lighten	Regression	Regression
Control group before treatment	Serious	Serious	Presence
Control group after treatment	Lighten	Invalid	Not subsided

## Data Availability

The data used to support the findings of this study are available from the corresponding author upon request.
